# Coordination Complexes as a New Generation Photosensitizer for Photodynamic Anticancer Therapy

**DOI:** 10.3390/ijms22158052

**Published:** 2021-07-28

**Authors:** Kacper Pobłocki, Joanna Drzeżdżon, Tomasz Kostrzewa, Dagmara Jacewicz

**Affiliations:** 1 Department of Environmental Technology, Faculty of Chemistry, University of Gdansk, Wita Stwosza 63, 80-308 Gdansk, Poland; kacperpoblocki@wp.pl (K.P.); dagmara.jacewicz@ug.edu.pl (D.J.); 2 Department of Medical Chemistry, Faculty of Medicine, Medical University of Gdansk, 80-211 Gdansk, Poland; tomasz.kostrzewa@gumed.edu.pl

**Keywords:** photodynamic therapy, ruthenium(II)-based complexes, iridium(III)-based complexes, osmium(0,II)-based complexes, copper(0,I)-based complexes, platinum(0)-based complexes, reactive oxygen species, photosensitizers, cancer therapy, reactive singlet oxygen

## Abstract

Photodynamic therapy (PDT) has become an alternative to standard cancer treatment methods such as surgery, chemotherapy and radiotherapy. The uniqueness of this method relies on the possibility of using various photosensitizers (PS) that absorb and convert light emission in radical oxygen-derived species (ROS). They can be present alone or in the presence of other compounds such as metal organic frameworks (MOFs), non-tubules or polymers. The interaction between DNA and metal-based complexes plays a key role in the development of new anti-cancer drugs. The use of coordination compounds in PDT has a significant impact on the amount ROS generated, quantum emission efficiency (Φem) and phototoxic index (PI). In this review, we will attempt to systematically review the recent literature and analyze the coordination complexes used as PS in PDT. Finally, we compared the anticancer activities of individual coordination complexes and discuss future perspectives. So far, only a few articles link so many transition metal ion coordination complexes of varying degrees of oxidation, which is why this review is needed by the scientific community to further expand this field worldwide. Additionally, it serves as a convenient collection of important, up-to-date information.

## 1. Introduction

**Figure 1:**
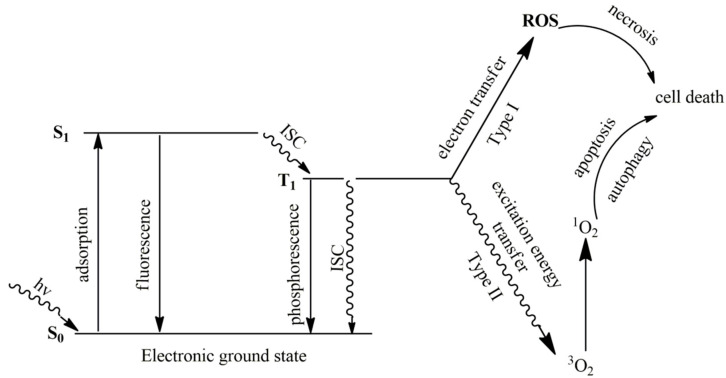
Modified Jabłonski’s diagram illustrating the principle of photodynamic therapy (ROS = reactive oxygen species). (cf to text for explanation). Adapted from [45] with permission from *Acc. Chem. Res.*, Copyright ACS 2017.

Worldwide, 70% of deaths are caused by cancer. According to the World Health Organization (WHO), obesity, ultraviolet radiation and infections: bacterial, viral or fungal contribute to the development of cancer [1]. Treatments such as chemotherapy, radiation therapy, and immunotherapy are being replaced by less invasive treatments such as photodynamic therapy (PDT). This innovative technique is attractive in terms of specificity and for not damaging healthy cells compared to other cancer treatments. Despite the rapid development of modern ways fighting cancer, classic methods such as chemotherapy remain the only option in the treatment of certain types of cancer, e.g., leukemia [2,3]. The first records of the healing properties of the Sun’s rays date back to ancient times. Heliotherapy, or exposing certain parts of the body to the Sun, has been used to cure vitiligo, rickets and psoriasis in Egypt, India, Rome and Greece. However, with the advent of Christianity and the fall of the Roman Empire, heliotherapy disappeared from the pages of history until the end of the 19th century. In 1898, Oscar Raab introduced photosensitizers (PS) in photodynamic therapy and his scientific research contributed to a sharp increase in interest in this method of treatment. Raab, Rikli and Finsen are considered the pioneers of modern photodynamic therapy. However, the first clinical data appeared in 1905. They were published by von Tappeiner and Jesionek, who used various dyes such as eosin or fluorescein as PS in the treatment of cutaneous lupus, melanoma and genital warts caused by HPV *(human papilloma virus*), which contribute to the development of cervical cancer. The research of Tappeiner and Jesionek is important because they were the first to report that oxygen was essential in photodynamic therapy [4]. The breakthrough moment for patients is believed to be 1960 when it was used by Finsen, when he was treating a rash caused by chickenpox [5]. It can be concluded from recent scientific publications that PDT has a positive effect on the treatment of dental caries by eradication of microorganisms and mycosis, i.e., *Candida albicans*, by combating biophylmes. Furthermore, PDT has been used in the imaging of atherosclerosis and in the treatment of condyloma [6,7,8,9]. Besides high selectivity and reproducibility, PDT is non-invasive. In most cases, it is a non-surgical therapy, thanks to which the patient does not have to undergo the procedure for a long time [9,10]. However, there are exceptions, and so far, the association of PDT with surgery has only been used in animals [10]. PDT activity is influenced by the duration of irradiation and the concentration of photosensitizers [11]. In PDT, in addition to the light source and PS, the proportion of oxygen is necessary. PS are photosensitive compounds capable of absorbing and transforming light energy as a consequence of creating an excited state. PS interacts with the environment in two ways. In type I, it is possible to generate reactive oxygen species (ROS) by reacting free radicals with oxygen. Radical ions are formed by transferring hydrogen atoms, formed as a result of reactions between proteins or lipids and PS’ excited triplet state. However, the more dominant type is II, in which the major oxidant molecule produced is singlet oxygen. It arises because of the action of the triplet excited state PS and oxygen in the triplet state. Both types occur at the same time [12]. PS can function independently, e.g., as photosynthetic pigments, chlorophylls and bacteriochlorophylls. The Fiedor research group has studied, inter alia, the effect of replacing magnesium in the center of bacteriochlorophyll with other metals such as zinc or copper [13,14,15,16]. On the other hand, photosensitizers can combine with MOFs, hydrogels, nanotubes or polymers [17]. Satisfactory features of the photosensitizer are the following: the ability to photothermal conversion in the near infrared range, addition of functional groups, the ability to photo-inactivate microorganisms and a large specific surface area. PDT applied on biofilm it is more effective in the case of bacteria with a porous structure of cell covers. Photosensitizers are then transported to the cytoplasmic membrane. An important issue is a balance between the hydrophobic and hydrophilic character of the PS molecule. For example, too much hydrophilic character leads to decreased membrane activity and transport, while too high hydrophobic character leads to better cellular uptake, but may lead to aggregation and reduction of ROS production. Scientists combat this problem by adding micellar, polymeric, or lysosomal compounds [10,18,19,20,21,22]. In the case of using PS against microorganism it must be noted that micellar or polymeric compounds such as Triton X-100, mPEG-b-p (HPMAm-Lac(2) and (Ce6)-loaded micelle system encapsulating cyanine dye (Cypate) (Cy/Ce6-Micelles) are frequently reported in the literature. In the case of using PS for cancer treatment it should be noted that the addition of such compounds to hydrophobic PS, e.g., Photofrin^®^ and Visudyne^®^, results in cell internalization, increases water solubility, increases cellular uptake and photostability. It allows one to get better quality and resolution for cellular imaging [14,23,24,25,26,27]. The use of the above-mentioned photosensitizers also has other disadvantages such as the problem of being excreted very slowly from the human organism. The patient undergoing photodynamic therapy is exposed to several weeks’ sensitivity to the Sun’s rays [28,29,30,31,32]. Most of the complex compounds show low absorption of radiation, therefore, when synthesizing new coordination compounds, ligands are carefully selected, or the existing complex compounds are skillfully modified. Effective photosensitizers can be defined as compounds showing strong absorption in the therapeutic window from 600 to 900 nm, thanks to which the cancer cells are penetrated deeper [33]. To start with photodynamic therapy, a photosensitizer (PS) in a specific concentration is delivered to the cancer cells. Then, once the PS is located in the tumor, the cancer area is irradiated with light of a specific wavelength. The PS molecule is excited because of absorption of energy irradiation. This energy is then transferred molecules to molecular oxygen (O_3_) in the vicinity of the tumor [33,34,35,36,37]. The reactive oxygen species, such as: singlet oxygen (^1^O_2_)_,_ hydroxyl radicals (HO^·^), superoxide radical (O_2**˙**_^−^), cause vascular damage, inflammation, and trigger neoplastic cells to cell death [38,39,40]. The entire scheme can be described using the Jabłoński diagram (Figure 1). Two energy levels should be distinguished. Singlet (lower energetic), into triplet (higher energetic), in which the spins of the electrons in the active state are directed parallel. When one of the electrons in the S_0_ sub-state absorbs energies, it is then excited into higher-energy molecular orbitals. Then it can return to the ground state (e.g., S_2_ => S_1_ or S_1_ => S_0_) through internal conversion, i.e., through the non-radiative transition of an electron of the same multiplicity, or through fluorescence, i.e., a radiant transition of an electron with the same multiplicity. On the other hand, there is also the possibility of an intercombination transition (ISC) between the singlet state and the much more permanent or triplet state (lifetime from micro to milliseconds). This is much more energetically advantageous because the lifetime of a singlet excited state is shorter (nanoseconds). At the triplet energy level, the same can also be happening to the singlet state described above. The electron at this energy level behaves in two ways. In the first, the photoinduced electron directly reacts with oxygen via charge transfer and the generation of ROS such as: hydrogen peroxide (H_2_O_2_), HO^·^, and O_2**˙**_^-^. On the other hand, the second method depends on the transfer of energy to molecular oxygen, which causes formation of highly reactive ^1^O_2_. Both free radicals and singlet oxygen are cytotoxic, lead to biological oxidation and can irreversibly destroy cancer cells [40,41,42,43,44]. The interaction of the hydroxyl radical with deoxyribose residues causes the formation of breaks in the DNA chains. ROS increase the amount of Ca^2+^ ions in cells, and also influence their release from cellular reserves. The increase in Ca^2+^ concentration activates ion-dependent endonucleases which degrade DNA. ROS cause the oxidation of amino acids with a free amino, amide or hydroxyl group, the consequences of which in proteins are the formation of crosslinking bonds.

In this paper, we have included a literature review of the coordination complexes most often used in cancer treatment as photosensitizers: ruthenium(II)-; iridium(III)-; osmium(0,II)-; copper(0,I)-; and platinum(II)-based complexes. We also present an approach to the problem of searching for new photosensitizers in the context of complex compounds.

## 2. Structure and Physicochemical Characteristics of Coordination Complexes as Photosensitizers for Photodynamic Anticancer Therapy

### 2.1. Ruthenium(II)-Based Complexes

**Figure 2:**
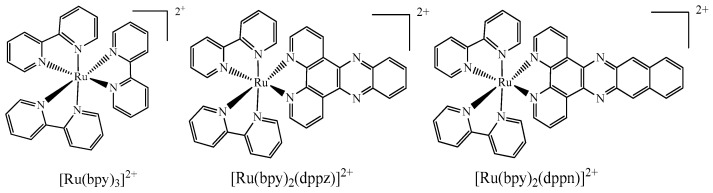
Chemical structure of [Ru(bpy)_3_]^2+^ (bpy = 2,2′-bipyridine), [Ru(bpy)_2_(dppz)]^2+^ (dppz = dipyrido[3,2-a:2′,3′c]phenazine) and [Ru(bpy)_2_(dppn)]^2+^ (dppn = benzo[i]dipyrido[3,2-a:2′,3′-c]phenazine). Adapted from [48] with permission from the journal *Chem. Sci*., Copyright 2015.

Compounds which, in addition to platinum, are frequently reported in publications on photosensitizers used in PDT are the Ru(II) complex compounds. Over the years, interest in organometallic compounds based on Ru(II) has increased due to their stable reaction kinetics, extensive physicochemical and electrochemical properties, low toxicity (non-toxic up to 100 μM for MRC-5 and HeLa) and specific transport within cells by transfer-catalyzing enzyme-specific chemical groups. Another advantage of these compounds are their ease of synthesis, facile modification of their topology, and thus different spatial and coordination geometries. An interesting phenomenon is “activation by reduction”, which consists in changing the oxidation state of Ru from Ru(III) to Ru(II) under the influence of the hypoxia usully found in tumors [45]. The Ru(II)-based parent compound [Ru(bpy)_3_]^2+^ (bpy—2,2′-bipyridine) (Figure 2) is the photophysical and photochemical reference for the rest of the ruthenium(II) compounds. At about 420 nm, excitation takes place and changes to the ^3^MLCT state (2.1 eV), which has a viability of 200 ns (in oxidized MeCN), 76 µs (in deoxygenated MeCN). In contrast, the quantum emission efficiency (Φem) and ^1^O_2_ formation are 10% (deoxygenated MeCN) and 56% (oxygenated MeCN), respectively. The second complex compound based on Ru(II) that we would like to characterize is [Ru(bpy)_2_(dppz)]^2+^ (dppz = dipyrido[3,2-a:2′,3′c]phenazine). Its uniqueness can be attributed, inter alia, to the presence of the dppz ligand, which has a positive effect on cellular uptake [45]. Other features of this complex compound are effective phototoxicity at 625 nm, despite a molar absorption coefficient of <100 M^−1^ cm^−1^ and its luminescence properties, which are attributed to the excited state of ^3^MLCT. Irradiation of metal-based photosensitizers with UV light results in photoactivation and exchange of photo-ligands with the formation of strong covalent bonds with biological systems, including DNA. The bond formation between the metallic-organic complex and DNA can be followed by spectroscopy. The photo-links can be seen from the additional absorption bands from the LF or excited state of MLCT visible in the spectrum. This is manifested, for example, by wide MLCT transitions in the light of the therapeutic window and high molar absorption. The photo-linkage derived from the low-energy state of the MLCT is attractive because it covers the visible light range. In addition, we can learn about important physicochemical aspects such as photostability and bond hydrolysis. On the other hand, not only can bonding be observed in the UV or fluorescence spectra, it is also important to be able to track the quenching of energy transfer or DNA photo-cleavage by ROS producing [25,46]. Therefore, [Ru(bpy)_2_(dppz)]^2+^ (Figure 2) is an efficient, molecular, luminescent DNA linker. It represents an extremely interesting example of a PS used in PDT therapy [47], due to the possible formation of ROS. For many years, little attention has been paid to subtle changes in the ligands in organometallic structures. However, scientists have recently investigated the differences between [Ru(bpy)_2_(dppz)]^2+^ and its analogue with the ligand dppn (benzo[i]dipyrido[3,2-a:2′,3′-c]phenazine). They concluded that the lifetime in the excited state is about 33 µs in MeCN for [Ru(bpy)_2_(dppn)]^2+^ (Figure 2), and about five times greater for the emission state lifetime of [Ru(bpy)_2_(dppz)]^2+^. A strong phototoxic effect is obtained through a prolonged triplet excited state. Long-lived 3IL excited states have been shown to produce ^1^O_2_ despite low oxygen pressures, therefore, they are defined as ideal candidates for use in photodynamic anti-cancer therapy [47]. Scientists have also investigated the effect of substituents on the phototoxicity, proving that the presence of the following ligands: 2,3-bis(2-pyridyl)pyrazine, 4,7-diphenyl-1,10-phenanthroline, 2,2′-bipyridine, 1,10-phenanthroline has a significant effect on photoactivity. The cervical cancer HeLa cells were treated with radiation at 420 nm and fluence corresponding to 9.27 J·cm^−2^. Satisfactory PI (phototoxic index) results above 150 were obtained for amino group-substituted [Ru(bpy)_2_(dppz)]^2+^ and PI 43 for methoxy group-substituted [Ru(bpy)_2_(dppz)]^2+^. The above-mentioned MLCT derivatives showed strong ruptures of plasmid DNA after exposure to light irradiation.

**Figure 3:**
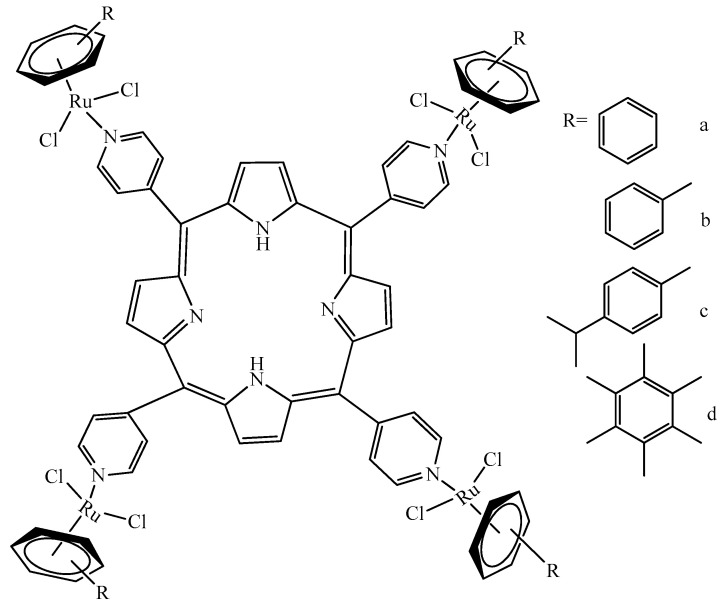
Chemical structure of Ru(II) based on meso-4′-tetrapyridylpyrin. Adapted from Ref. [48] with permission from the *Chem. Sci.*, Copyright 2015.

Recently, scientists have achieved high anti-cancer effects by modifying the porphyrin structure with benzene groups and its derivatives. The research group led by Therrien added several Ru(II)-based arene fragments to the meso-4′-tetrapyridylpyrin scaffold to assess the impact of aromatic moieties (Figure 3). They showed that all the compounds listed below showed 60–80% cytotoxicity on human Me300 melanoma cells. The studies have been carried out at a wavelength of 652 nm (to ensure the deepest possible penetration of cancer cells) and a concentration of 10 μM and a fluence at 5 J·cm^−1^ (at 488 nm, power of 50 mW).

**Figure 4:**
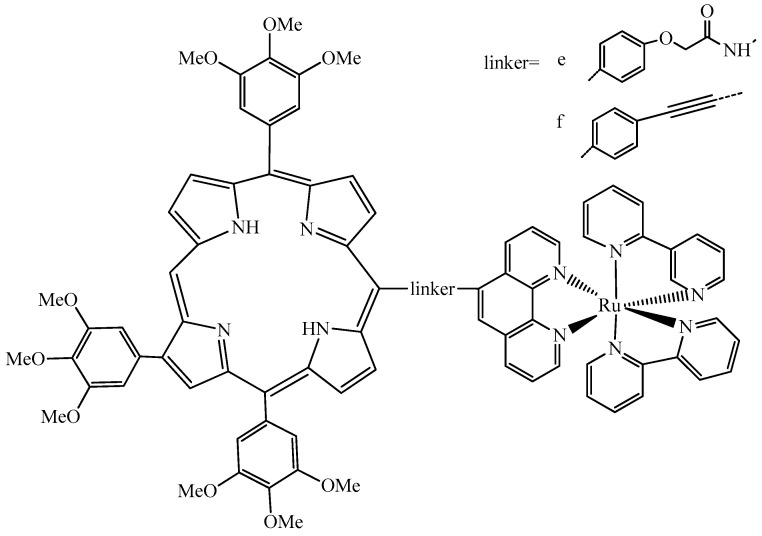
Chemical structure of [Ru(bipy)_2_(phen)]^2+^ (phen = 1,10-phenanthroline, Me = methyl group) linked to a porphyrin moiety. Adapted from [48] with permission from the journal *Chem. Sci*, Copyright 2015.

It was also shown that the phototoxic effect changes depending on the isomer used. Trisubstituted pyridylpyrine systems exhibit stronger phototoxic properties than four-substituted analogs. The last Ru(II)-based compound discussed by us will be [Ru(bipy)_2_(phen)]^2+^ (phen-1,10-phenanthroline) linked to a porphyrin moiety (Figure 4). The what? research group focused on the use of two different connectors, which made it possible to identify changes in the location of cancer cells. It has been proven that the compound is excited at 800 nm, which allows the light beam to penetrate deeper and damage cancer cells. The results of the studies on the compounds with linkers **e** (118 μM) and **f** (175 μM) showed the highest phototoxicity at the flow of 6.5 J·cm^−2^ for **e** and 2.0 J·cm^−2^ for **f** on cervical cancer cells (HeLa) using 850 nm radiation and 8 mW power. In addition, the compound with linker **f** changed its localization in a cell after irradiation, therefore it is assumed that placing it in the cytosol will result in membrane damage and subsequent cell death [45,46,47,48].

Collectively, in the case of the ruthenium(II) complex compounds used as PS, it should be emphasized that a special achievement is the design of a complex with selective photoactivation, which is a factor in selective treatments in anti-cancer therapy.

Designing new complex compounds as photosensitizers should be closely related to the so-called “heavy atom effect”. Due to the presence of the spin-orbital coupling, the possibility of an inter-system transition is increased. The so-called “heavy atom effect” affects the quantum yield. A low quantum yield of fluorescence indicates the possibility of an effective intersystem transition in these molecules, and thus the possibility of efficient generation of singlet oxygen by these compounds.

### 2.2. Iridium(III)-Based Complexes

**Figure 5:**
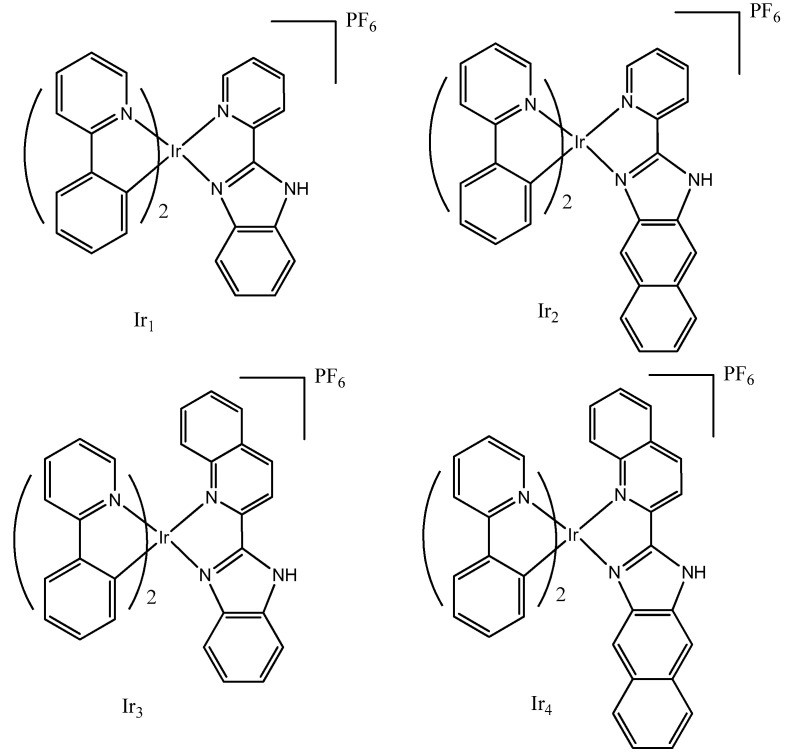
Chemical structures of complexes Ir_1_–Ir_4_. Adapted from [55] with permission from the journal *ACS Appl. Mater. Interfaces*, Copyright 2017 American Chemical Society.

The phosphorescent emission at 405 nm for the four benzimidazole-containing iridium(III) complex compounds (Figure 5) [49,50,51] may be due to an electron transition between ^1^MLCT (singlet ligand-to-ligand charge transfer) and ^3^MLCT in the ligands. The relationship between the number of aromatic rings with nitrogen atoms and a greater shift in emission was demonstrated. The greater number of rings reduces the electron deficit and stabilizes the lowest free molecular orbital (LUMO) [52,53,54,55]. An example where the greatest shift in radiation emission occurs is benzimidazole-containing iridium(III) complex compound [53,54,55].

The phototoxicity of all iridium(III)-based complexes was tested with cervical and lung cell lines after irradiation at 425 nm (Table 1). It ranges from 0.21 to 1.43 μM. It is worth emphasizing that Ir_3_ and Ir_4_ show higher phototoxicity (PI) and that iridium(III)-based photosensitizers like NAMI-A (containing Ru) inhibit the process of neoplastic metastasis from 25.1% to 29.4% at a fluence of 10 μM. The research of Wang’s team has shown that iridium(III)-based organometallic complexes not only inhibit the growth of cancer cells, but also the migration of cancer cells, colony formation and apoptosis of cancer cells by reactive oxygen species. The phosphorescent emission (at 405 nm) for the four iridium(III) complex compounds containing benzimidazole may be due to an electron transition between ^1^MLCT and ^3^MLCT in organic ligands. The relationship between the number of aromatic rings and nitrogen atoms in PS and a greater shift in emissions was demonstrated. A greater number of benzene or pyridine groups reduces the electron deficit and stabilizes the lowest free molecular orbital (LUMO). An example where the largest shift in radiation emission occurs is compound **4**. It is also worth noting that the quantum yield of ^1^O_2_ was much higher at acidic pH than in neutral or alkaline solutions [55].

The Ir(III)-peptide-based potential medical candidates provide an opportunity to improve the cancer selectivity. The use of easily breakable bonds in the synthesis of iridium(III) complexes improves the pharmacological properties of these photosensitizers [52,53,54]. The disadvantageous effect of the complex compounds used as photosensitizers is minimized by targeting only the diseased cells and only after the activation of the complexes. A common undesirable effect of therapy is erythema at the irradiation site, but today it is more and more eliminated by the use of conjugates in the production of photosensitizers. After therapy, the complex compounds are metabolized. Their possible adverse effects and side effects are dictated solely by their toxicity as preparations before activation and it depends on the specificity used. It is worth emphasizing that in comparison with other non-invasive anti-cancer therapies, the use of photosensitizers does not damage the bone marrow, as well as liver or kidney function [53,54,55].

### 2.3. Osmium(0,II)-Based Complexes

**Figure 6:**
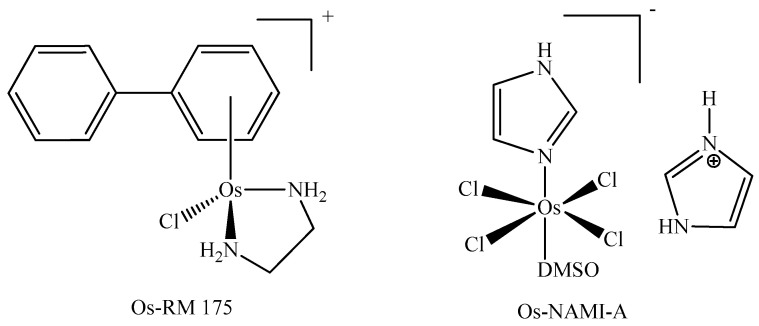
Chemical structures of the complexes Os-RM175 and Os-NAMI-A (DMSO = dimethyl sulfoxide). Adapted from [62] with permission from the journal *RSC Adv.*, Copyright © 2019.

Osmium(II) complexes show better properties as photosensitizers than platinum(II) complexes, because they have a high spin-orbit coupling constant, and thus electronic transitions from the ground state to excited states are more possible. Osmium(II) complexes combined with polyarginine are also known, which have proved successful in cellular uptake and imaging. Initially, osmium-based compounds were considered analogs of platinum anticancer agents [56,57,58]. The following osmium(II) complexes [Os(*N*^*N*)_3_]^2+^ (*N*^*N* = 1-benzyl-4-(pyrid-2-yl)-1,2,3-triazole; 1-benzyl-4-(pyrimidin-2-yl)-1,2,3-tria-zole and 1-benzyl-4-(pyrazin-2-yl)-1,2,3-triazole exhibit activity in PDT. After some time, their unconventional action, i.e., targeting targets other than DNA, was discovered. For example, the osmium analog NAMI-A is more inert and stable in an aqueous environment than NAMI-A alone. In vitro cytotoxicity tests showed that Os-NAMI-A (Figure 6) showed better properties than the original compound. Namely, it turned out that the osmium analog has three times higher activity against colon cancer cells (*HT-29*) and shows twice as much anti-tumor activity in breast cancer cell lines (*SK-BR-3*) compared to NAMI-A. In addition, the osmium analog was not overhydrated and was active against colon (*HT29*), lung (*A549*) and breast (*T47D*) tumor cells. Another RM175 analog, i.e., AFAP5151, showed a 40 times lower rate of hydrolytic degradation and a lower pH than RM175 (Figure 6). An interesting phenomenon was the decreased reactivity to 9-ethylguanine [59,60,61,62]. AFAP51 was able to bind DNA in cell-free media and was cytotoxic in the ovarian cancer cell line *A2780* [60]. The results have been obtained after irradiation with light.

Osmium(0,II)-based complexes are successfully used in photothermal therapy with high reactive species production [60,61,62].

### 2.4. Copper(0,I)-Based Complexes

Copper-cysteamine (Cu-Cy) nanoparticles are promising photosensitizing (PS) agents [63,64] that can be efficiently activated by X-rays to produce singlet oxygen for cancer therapy. They can be activated by various sources of excitation, such as UV radiation, microwaves (MW), X-rays and ultrasound (US), chemical compounds such as hydrogen peroxide or an acidic environment. Cu-Cy nanoparticles show strong luminescence, which is an interesting phenomenon because most of the copper-based complexes do not exhibit luminescent properties at all. There is intrinsic conversion inside the Cu-Cy molecule and therefore it produces very small amounts of ROS. Additionally, it was shown that large-sized Cu-Cy nanoparticles produce the lowest amount of ROS. Moreover, the amount of oxygen species generated is limited by such factors as particle shape, size, surface charge, solubility, physical state and spatial structure. Research has shown that smaller nanoparticles fluoresce less. This is due to the fact that if energy with a lower radiation range is applied to a nanoparticle with dimensions of about 40 nm, the volume of cancer cells decreases. 40 nm Cu-Cy nanoparticles, under the influence of X-ray stimulation, effectively inhibit the growth of melanoma cells. This is likely due to the larger specific surface area and much greater production of reactive oxygen species, which increases cellular uptake. On the other hand, medium-sized nanoparticles (about 100 nm), despite producing the largest amount of ROS among the examples mentioned, did not show anti-tumor activity against B16F10 melanoma cells both in in vitro and in vivo tests. However, scientists also conducted both in vitro and in vivo tests that confirmed the anti-tumor activity of Cu-Cy nanoparticles on SW620 colon cancer cells located at the orthotropic site [65]. Cu-Cy nanoparticles coupled with pHLIP show increased radiation effect and decreased volume. This confirms that Cu-Cy nanoparticles may be able to increase the efficiency of the PDT process in combination with pHLIP [66,67].

It is worth mentioning that complexes based on copper are successfully used in chemodynamic therapy and act similarly to the osmium(0,II) complexes by intensively generating ROS.

### 2.5. Platinum(II)-Based Complexes

**Scheme 1:**
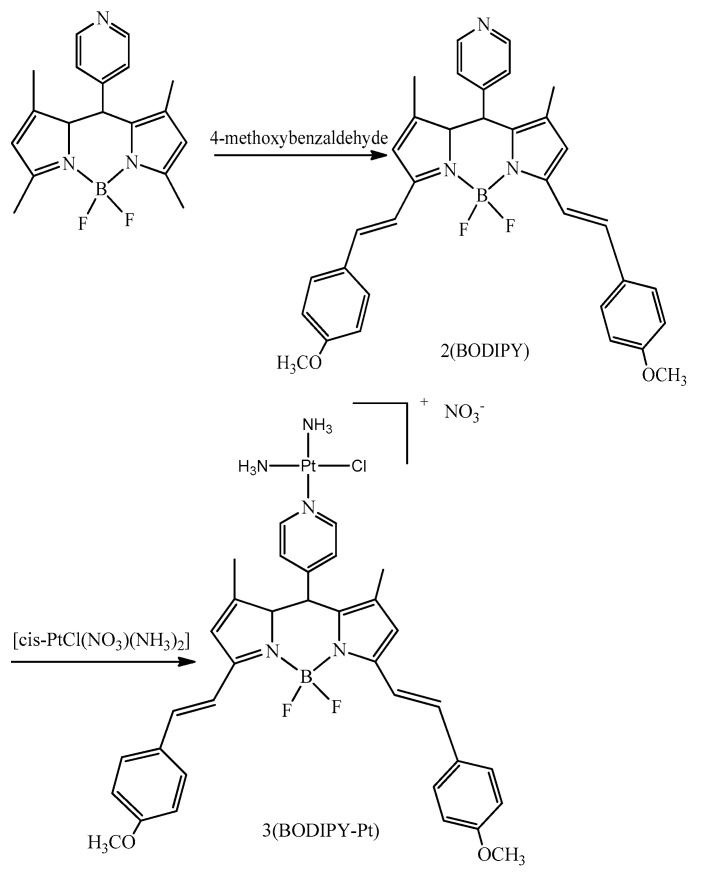
The synthesis procedure of BODIPY-Pt (BODIPY = (4,4-Difluoro-1,3,5,7,8-Pentamethyl-4-Bora-3a,4a-Diaza-s-Indacene). Adapted from [70] with permission from the journal *ACS Med. Chem. Lett.*, Copyright 2015 American Chemical Society.

The last coordination compound discussed will be BODIPY-Pt (Scheme 1) [68,69]. BODIPY-labeled Pt compound is synthesized by a simple and easy method. The results of the research confirmed that BODIPY-Pt accumulates mainly in the mitochondria. Additionally, it is worth emphasizing that the cellular uptake of BODIPY-Pt depends on the potential of the mitochondrial membrane. The cytotoxicity of BODIPY-Pt conjugates is slightly lower than that of cisplatin—a known anticancer drug. It has been proven that BODIPY-Pt can play a cytostatic role. Cellular uptake and imaging of BODIPY-Pt were studied in HepG2 cells residing in the cytoplasm of HepG2 cells. The quantum yield of the fluorescence of the coordination compound on platinum base (0) was (0.08), while BODIPY itself was (0.13). However, the cellular uptake of BODIPY-Pt was significantly increased as confirmed by flow cytometry. A possible reason for the increased uptake is that the introduction of platinum significantly increased the photodynamic activity of the entire complex. HepG2 and HeLa cancer cells were incubated with different concentrations of BODIPY-Pt, the results indicated that the IC_50_ value of BODIPY-Pt was 35.29 µM (HepG2) and 10.89 µM (HeLa). Comparing this with the cytoactivity of *cis*-platinum, we can conclude that despite the lower anti-tumor activity, we see a comparable relationship between concentration and cytotoxicity, which is a good prognostic. In short, we can conclude that BODIPY-Pt shows excellent phototoxicity, comparable to cisplatin [70,71]. Collectively, it is worth emphasizing that the use of a glucose group in ligand modification increases the solubility of the complex and thus contributes to the increase in cellular uptake.

## 3. Anti-Cancer Activities Comparison of Ru(II), Ir(III), Os(0,II), Cu(0,I), Pt(II) Coordination Complexes in Photodynamic Anticancer Therapy

Comparing anti-cancer activities between different coordination compounds is difficult. One should start with the use of the same ligands in all compounds [71]. However, compounds based on ruthenium(II) show the strongest anti-cancer properties. They are widely described in the literature and in our opinion other coordination structures should be compared to complexes based on Ru(II). A group of compounds that can inhibit several key neoplastic events including cell migration, invasion, colony formation, and *in vivo* angiogenesis are described above by Ir_1_-Ir_4_ [54,55]. Therefore, their pH-dependent phosphorescence exhibit increased emission in lysosomes. After low-energy irradiation, Ir_1_–Ir_4_ can initiate cancer cell apoptosis through ROS upregulation, caspase activation and lysosomal damage [54,55]. Ir(III) complexes typically exhibit shorter absorption wavelengths than the reported Ru(II) complexes. Moreover, some rigid heteroleptics and most homoleptics of iridium(III)-based coordination complexes are less soluble in aqueous medium than Ru(II) complexes. Appropriate structural modifications are likely to overcome these drawbacks [72,73,74,75]. On the other hand, PS based on Os(II) show a character indifferent to the ligand substitution compared to Ru(II) and therefore often causes a reduction in hydrolysis, breaking the metal-ligand bonds. Moreover, after hydrolysis, the formed Os-aqua species show a more acidic pH than the corresponding Ru-aqua species (i.e., decrease in pKa by 1.5 pH units). Consequently, if hydrolysis occurs, the Os(II) complexes form non-reactive hydroxide species under physiological conditions. On the other hand, a hypoxic and slightly acidic tumor may bind water complexes. According to the HSAB principle, Os(II) is softer than ruthenium, which is likely to result in slightly different coordination preferences for biomolecules [75,76]. The remaining organometallic complexes with zero oxidation state do not show significant anti-cancer properties. In addition, there are very few publications on them, which is a great field for research teams around the world to work with.

## 4. Designing New Complexes Increasing the Efficiency of Photodynamic Therapy in Multimodal Oncology

Special attention should be paid to the design of organo-based photosensitizers that can be excited by radiation with nanoplatforms, such as nanoparticles, to facilitate their in vivo and in vitro applications [5]. Moreover, the commercially available first-generation photosensitizer, Photofrin^®^, has some limitations in clinical use, such as relatively poor tissue selectivity and low light absorption. Therefore, it is still a big challenge to develop second-generation photosensitizers with high PDT activity, and also without side effects [76,77,78,79]. The electronic properties of TiO_2_ allow the PS to adhere to its surface, increasing the TiO_2_ absorption profile, which allows the use of visible light, more effectively inactivating bacteria, human keratinocyte (*HaCaT)* and prostate cancer cells (*DU145*). To overcome the current limitations of PDT, new synergistic treatments have been adopted which combine PDT with other therapies such as photothermal therapy (PTT) [79,80]. For example, nanoparticles (NPs) such as carbon nanorods are used with gold compounds (GV) 14 and graphene oxide which increases tumor PS accumulation and the production of heat and singlet oxygen for synergistic PDT/PTT. However, PDT/PTT based on photothermal coupling agents generally requires two lasers with different wavelengths due to the absorption mismatch between the PS and the photothermal agents. Sequential irradiation prolongs treatment and requires precise alignment of the two light beams. Therefore, there remains a great challenge to: develop a simple and effective therapeutic strategy for the simultaneous synergistic treatment of PDT/PTT. For example, NIR dyes are used as promising imaging and therapeutic agents in the treatment of tumors with PTT or PDT. The second example is indocyanine green (ICG) is a tricarbocyanine NIR dye with a maximization of absorption and emission around 780 and 830 nm in the NIR region with low absorption by tissue chromophores. However, ICG’s therapeutic use is limited by various factors, such as poor in vitro aqueous stability, concentration-dependent aggregation behavior, short circulating half-life, and effects beyond [78,79,80,81]. Recent advances in the discovery of Ir(III) photosensitizers could: lead to a new generation of PDT agents. The attractiveness of photoactive coordination compounds on iridium(III) base arise from their tunable photochemical and photophysical properties. The photobiological and photochemical activity of Ir(III) photoactive complexes, including cellular uptake, subcellular localization, excitation wavelengths, their emission and ROS production can be finely tuned by appropriate modification of the ligands. A small structural change can result in a major change in the target tissue, for example in cell organelles or proteins.

Recent efforts have led to the development of Ir(III) photosensitizers with excitation wavelengths in the range of 600–900 nm, which provides anti-tumor performance. Excitation in this near infrared region can be achieved by means of Ir(III) photosensitizers excited by two photons. The condition for using the two-photon method is that the compound must absorb two photons, and then the complex can generate singlet oxygen in the presence of atmospheric oxygen. In the case the complexes do not absorb then chromophores are used. However, the high cost and less convenient use of two-photon light, along with a lack of in vivo efficiency, must be addressed in the future. Moreover, it is clear that recent research into PS based on Ir(III) has begun to take a new path in PDT photochemotherapy thanks to its clinical development potential [72].

## 5. Conclusions and Future Perspectives

PDT is a very attractive medical technique due to its intrinsic selectivity. The results of PDT treatment depend on the action of PS, but also on other very important factors (e.g., light wavelength, the amount of reactive oxygen species formed, etc.). A candidate chemical compound for the role of a good photosensitizer should meet the following features: be chemically pure, have the property of accumulation only in strictly defined diseased cells, have an absorption spectrum in the range of very deep penetrating radiation, destroy target cancer cells, not be toxic to the human oragnism. ROS is formed at the site of light irradiation, with full spatial and temporal control. They are harmless on their own but in combination with PS they exhibit toxicity. Another advantage is the presence of rapidly reactive ^1^O_2_ which only damages limited areas.

The following years will bring progress in explaining the effectiveness, mechanisms and activities that will provide a solid support for further development in in vitro and in vivo research. Contributions to the development of screening tests as well as models that mimic human physiology should be made to make the use of PS based on coordination compounds more common in the future. The main challenge in PDT is the development of PS molecules that absorb light in the phototherapeutic window in the 600–900 nm range—where human tissues are most susceptible to the action. It is also important to improve the elements related to the excretion of PS from the body, e.g., treatment with Photofrin^®^ results in several weeks of photosensitivity due to the slow removal of the drug from the human organism. In addition, it shows poor selectivity, low light absorbance and side effects. Future work should focus on reducing the disadvantages (side effects) present in PS use such as the use of hydrolysable monodentate ligands (such as chloride), selection of counter-anions, charge balance by selecting the appropriate ligand. The last thing worth mentioning is hypoxia. Cancer cells suffer from severe hypoxia that greatly reduces the effectiveness of PDT. In addition, PDT, which consumes oxygen, will further aggravate tumor hypoxia and thus lead to many undesirable consequences such as angiogenesis, invasiveness and tumor metastasis. Tumor hypoxia should be used to increase the therapeutic efficacy of PDT.

New complex compounds which are candidates for the role of effective photosensitizers in PDT should contain modified auxiliary ligands with specific functional groups so as to thus control cellular uptake and biocompatibility. PS must strongly absorb radiation in the 600–900 nm range which penetrates deeply into the tissues and it is less limited by the endogenous absorption of water and dyes. Designing new PS should be based on knowledge about structure and photophysical properties relationship. It should be noted that such ligands as phthalocyanine and naphthalocyanine groups, which show very advantageous photophysical properties. Additionally, the modification of ligands with linkers determining anti-tumor activity should be taken into account. It is absolutely essential to carefully analyze the structural parameters influencing the pharmacokinetics and pharmacodynamics of the complex compounds when designing new PS. Most of the currently available results of research on complex transition metal compounds as photosensitizers are preclinical. Therefore, there are still many challenges faced by scientists to ensure that the proposed complex compounds meet the requirements of clinical trials.

## Figures and Tables

**Table 1:** Cytotoxicity and phototoxicity of complexes Ir_1_–Ir_4_, cisplatin and NAMI-A against cervical cancer (HeLa) and lung cancer (A459), (A549R) [55].

		HeLa		A459		A549R	
Complex		IC_50_ (μM)	PI ^a^	IC_50_ (μM)	PI	IC_50_ (μM)	PI
**Ir_1_**	Dark	11.69	8.4	7.64	8.0	5.98	4.2
**Ir_1_**	Light ^b^	1.39		0.95		1.43	
**Ir_2_**	Dark	15.13	63	5.76	24	4.26	9.7
**Ir_2_**	Light	0.24		0.24		0.44	
**Ir_3_**	Dark	>100	>200	>100	>137	>100	>83
**Ir_3_**	Light	0.50		0.73		1.20	
**Ir_4_**	Dark	>100	>476	>100	>322	>100	>139
**Ir_4_**	Light	0.21		0.31		0.72	
**Cisplatin**	Dark	12.04	1.1	8.57	1.0	73.42	1.0
**Cisplatin**	Light	10.56		8.42		69.38	
**NAMI-A**	Dark	>100		>100		>100	
**NAMI-A**	Light	>100		>100		>100	

^a^ PI (phototoxic index) is the ratio of the IC_50_ value in the dark to that obtained upon light irradiation. ^b^ Cells were irradiated by a 425 nm LED light array with 1.2 Jcm^−2^ (4 mW·cm^−2^, 300 s) after incubation with the tested complexes for 12 h. Adapted from [55] with permission of the journal *ACS Appl. Mater. Interfaces*, Copyright 2017 American Chemical Society.

## Data Availability

Not applicable.
